# Supplementary material for: Pseudomonas aeruginosa SutA wedges RNAP lobe domain open to facilitate promoter DNA unwinding
Source: Nat Commun. 2022 Jul 20;13:4204. doi: 10.1038/s41467-022-31871-7 (PMC9300723; doi:10.1038/s41467-022-31871-7)
Supplement: Supplementary file 1 — Supplementary Information [file 41467_2022_31871_MOESM1_ESM.pdf]

***Pseudomonas aeruginosa* SutA wedges RNAP lobe domain open to facilitate promoter DNA unwinding**

Dingwei He *et al*.

Supplementary Table 1. Plasmids used in this study

Plasmids	Source
pET-28a-TEV	Gift
pET-28a-TEV-SutA (WT)	This study
pET-28a- TEV-SutA (ΔN)	This study
pET-28a- TEV-SutA (ΔC)	This study
pET-28a-TEV-σ^S (WT)	This study
pET-26b	Gift
pET-26b-σ^S (WT)	This study
pET-26b-σ^A (WT)	This study
pET-26b-$\sigma^S_{1.1}$ (A2C)	This study
pET-26b-$\sigma^A_{1.1}$ (S2C)	This study
pET-26b-σ^A (Δ1.1)	This study
pGADT7 SutA (1-105)	This study
pGADT7-SutA (56–105)	This study
pGADT7-SutA (56–90)	This study
pGADT7-SutA (1–55)	This study
pGADT7-SutA (1–79)	This study
pGADT7-SutA (56–79)	This study
pGADT7-SutA (80–105)	This study
pGADT7-SutA-RBD (S56A)	This study
pGADT7-SutA-RBD (K60A)	This study
pGADT7-SutA-RBD (R64A)	This study
pGADT7-SutA-RBD (L67A)	This study
pGADT7-SutA-RBD (M71A)	This study
pGADT7-SutA-RBD (E72A)	This study
pGADT7-SutA-RBD (F74A)	This study
pGADT7-SutA-RBD (L75A)	This study
pGADT7-SutA-RBD (G78F)	This study
pGADT7-SutA-RBD (G79F)	This study
pGADT7-SutA-RBD (V81A)	This study
pGADT7-SutA-RBD (E83A)	This study
pGADT7-SutA-RBD (I84A)	This study
pGBKT7-β protrusion	This study
pCOLA-*Pae rpoB-rpoC*	This study
pACYC-*Pae* rpoA-rpoZ	This study
pEasyT-*rrn*-Mango	This study

Supplementary Table 2. The statistics of cryo-EM structure determination of *Pae* RNAP-σ^S-holoenzyme, *Pae* SutA-σ^S-RNAP (open lobe), SutA-σ^S-RNAP (closed lobe), and *Pae* SutA-σ^S-RPo.

	***Pae* RNAP-σ^S**	***Pae* SutA-RNAP-σ^S (open lobe)**	***Pae* SutA-RNAP-σ^S (closed lobe)**	***Pae* SutA-σ^S-RPo**
**Data collection**				
Number of grids used	1	1	1	1
Grid type	Holey carbon	Holey carbon	Holey carbon	Holey carbon
Microscope	Titan Krios	Titan Krios	Titan Krios	Titan Krios
Detector	Gatan K2	Gatan K3	Gatan K3	Gatan K2
Voltage (keV)	300	300	300	300
Dose rate (e^-/s)	10.0	22.5	22.5	8.0
Pixel size (Å/pix)	1.307	1.100	1.100	1.000
Total dose (e^-/ Å^2)	58.5	50.0	50.0	60.80
Total exposure time (s)	10	2.67	2.67	7.6
Number of Frames/movie	40	40	40	38
Defocus range (μm)	-1.8 to -2.6	-1.2 to -2.5	-1.2 to -2.5	-1.2 to -2.2
Number of micrographs	1080	2805	2805	2005
Particles used for final map	105,629	138,109	29,531	112,959
**Model composition**				
Non-hydrogen atoms	24247	25700	24560	25722
Protein residues	3425	3480	3479	3466
Nucleotide	N/A	N/A	N/A	78
Ligands (Zn2+/Mg2+)	2/1	2/1	2/1	2/1
**Refinement**				
Resolution (Å)	4.05	3.13	3.86	5.77
Sharpening B factors (Å^2)	-122	-92	-73	-154
Clash score	3.71	4.77	4.11	6.05
Average B factor (Å^2)				
Protein	43.2	56.9	47.7	215.3
Nucleotide	N/A	N/A	N/A	318.1
Ligand	41.2	50.8	33.6	181.5
RMS deviations				
Bond lengths (Å)	0.001	0.001	0.001	0.003
Bond angles (°)	0.360	0.366	0.366	0.734
Ramachandran plot				
Favored (%)	98.50	97.11	98.61	96.19
Allowed (%)	1.50	2.89	1.39	3.75
Outliers (%)	0	0	0	0

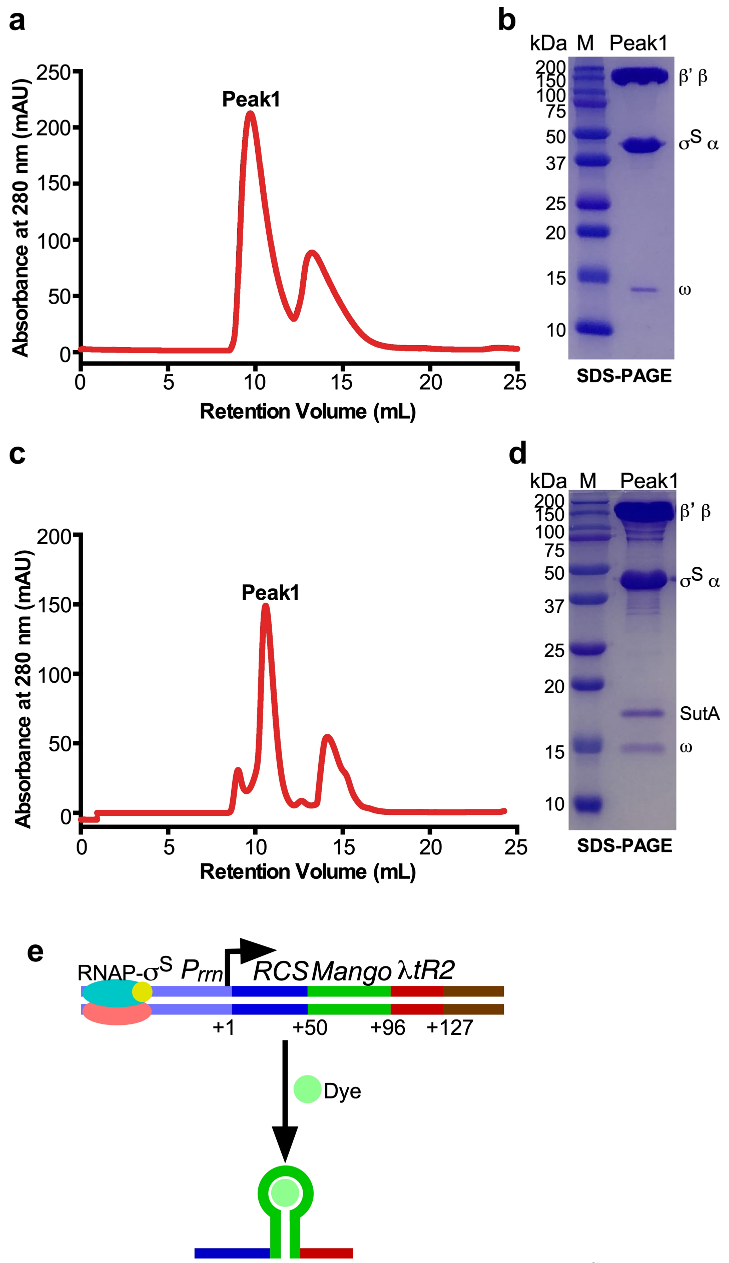

Supplementary Figure 1. The reconstitution of *Pae* RANP-σ^S and *Pae* SutA-RANP-σ^S complexes. a The size exclusion chromatography result shows the separation of RANP-σ^S holoenzyme (Peak 1). **b** The SDS-PAGE result of peak1. The experiment was repeated more than four times. **c** The size exclusion chromatography shows the separation of SutA-RANP-σ^S complex (Peak 1). **d** The SDS-PAGE result of peak1. The experiment was repeated more than four times. **e** The schematic of fluorescence-based in vitro transcription assay. The DNA template contains the *rrn* promoter (P_{rrn}), *rrn*-coding sequence (*RCS*), Mango III-encoding sequence (*Mango*; green), followed by a λt$_{R2}$ terminator (Red). +1, transcription start site; +127, transcription termination site.

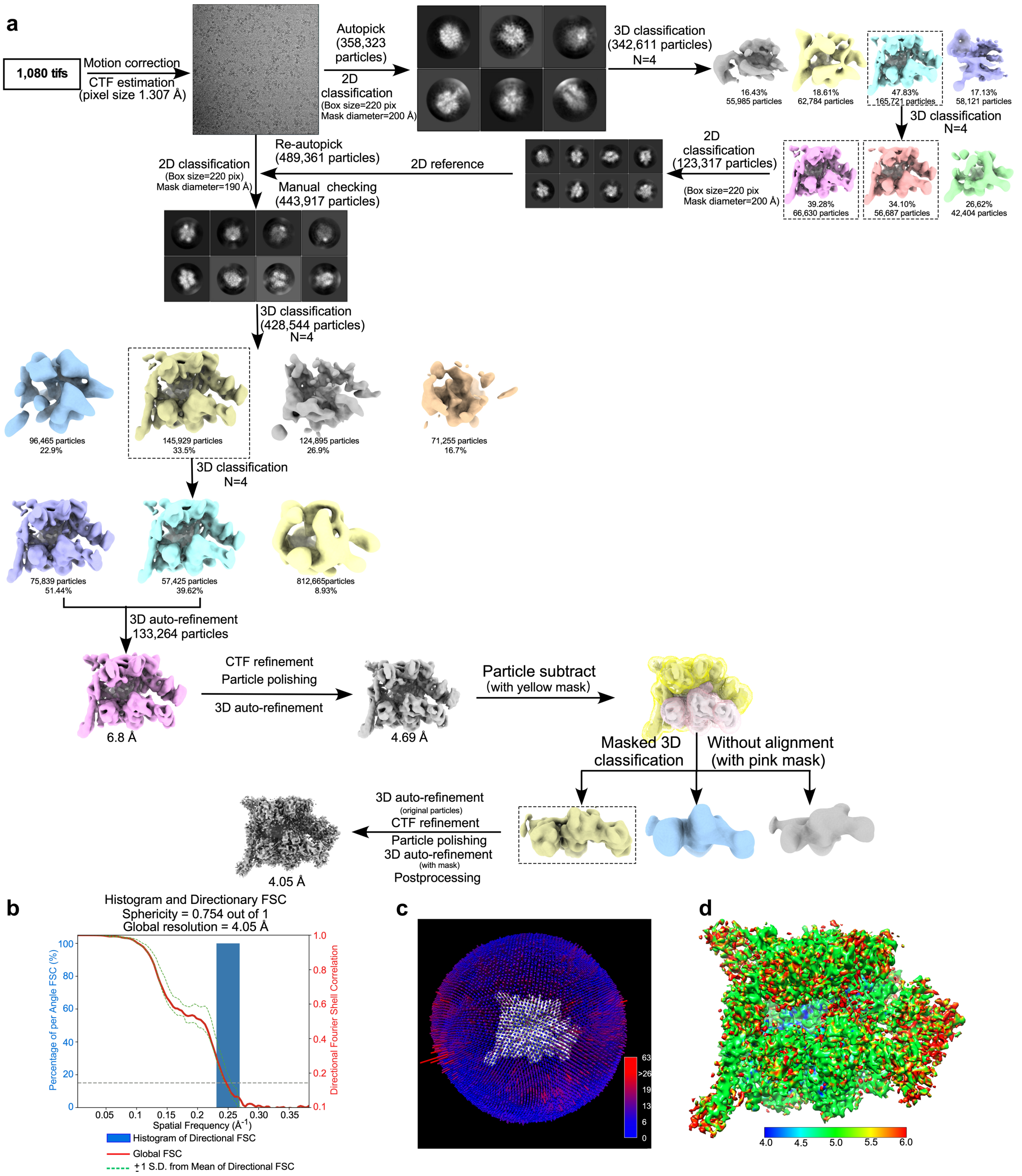

Supplementary Figure 2. The processing pipeline for cryo-EM map construction of *Pae* RNAP-σ^S holoenzyme. a The flowchart of cryo-EM data process for RNAP-σ^S holoenzyme**. b** The 3D FSC plot of RNAP-σ^S holoenzyme dataset. The dotted line represents the 0.143 FSC cutoff indicating a nominal resolution of 4.05 Å. **c** The angular distribution of particle projections. **d** The distribution of local resolution.

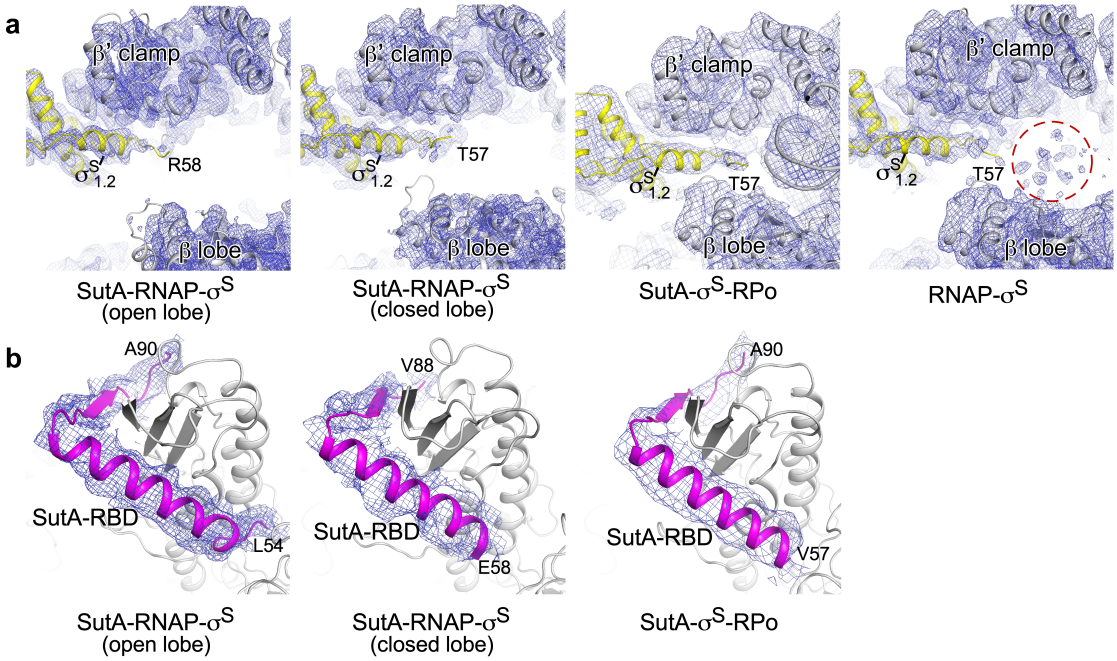

Supplementary Figure 3. Comparison of the weighted density map for $\sigma^S_{1.1\text{-}1.2}$ and SutA. **a** Side by side comparison of the weighted density map at the same contour level for $\sigma^S_{1.1\text{-}1.2}$ in SutA-RNAP-σ^S (open lobe), SutA-RNAP-σ^S (closed lobe), SutA- σ^S-RPo, and RNAP- σ^S holoenzyme. The weak fractionated map signals of $\sigma^S_{1.1}$ are labeled in red dashed circle. **b** Side by side comparison of the weighted density map for SutA at the same contour level in SutA-RNAP-σ^S (open lobe), SutA-RNAP-σ^S (closed lobe), and SutA- σ^S-RPo complexes.

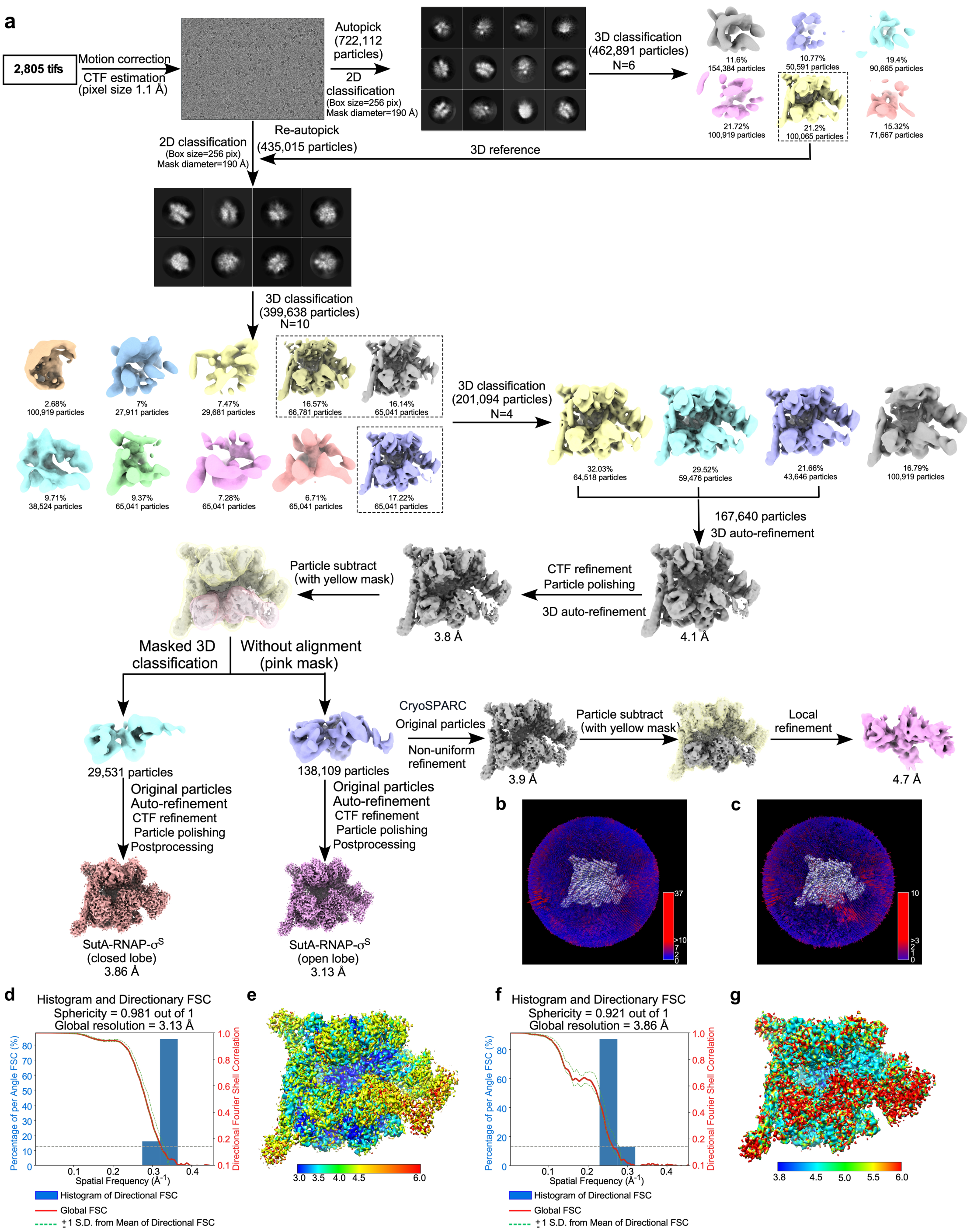

Supplementary Figure 4. The processing pipeline for cryo-EM map construction of *Pae* SutA-RNAP-σ^S complexes. a The flowchart of cryo-EM data process of SutA-RNAP-σ^S complex**. b** The angular distribution of SutA-RNAP-σ^S (open lobe) single-particle projections colored by number of particles of each projection. **c** The angular distribution of SutA-RNAP-σ^S (closed lobe) single-particle projections colored by number of particles of each projection. **d** The 3D FSC plot of SutA-RNAP-σ^S (open lobe) dataset. The dotted line represents the 0.143 FSC cutoff indicating a nominal resolution of 3.13 Å. **e** The local resolution of SutA-RNAP-σ^S (open lobe). **f** The 3D FSC plot of SutA-RNAP-σ^S (closed lobe) dataset. The dotted line represents the 0.143 FSC cutoff indicating a nominal resolution of 3.86 Å. **g** The local resolution of SutA-RNAP-σ^S (closed lobe).

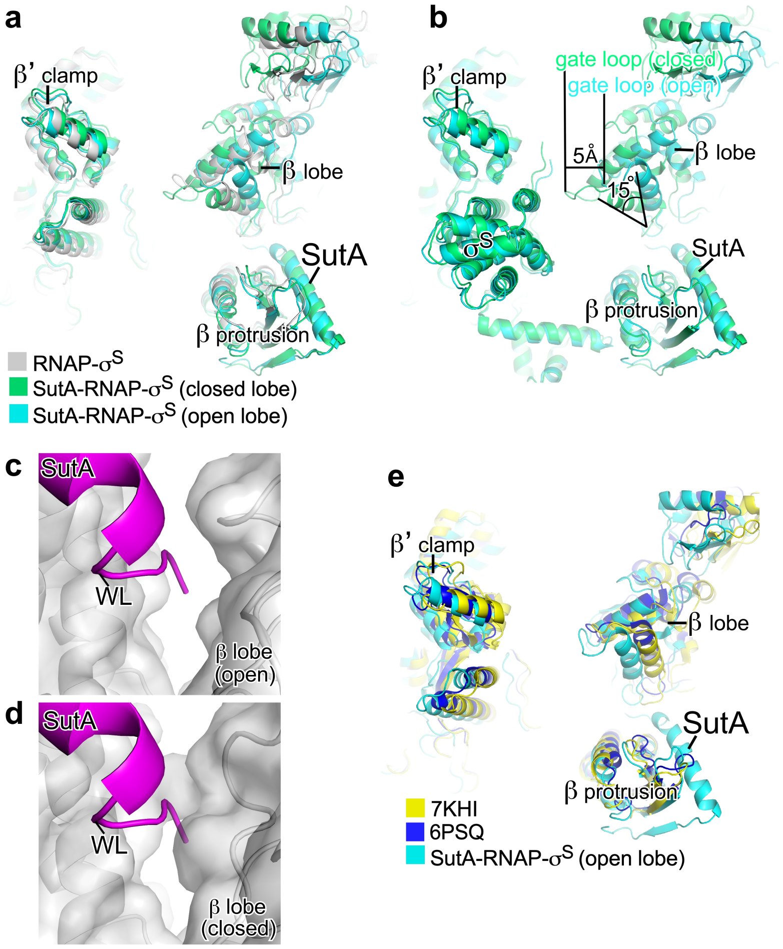

Supplementary Figure 5. The structural analysis of *Pae* RANP-σ^S-holoenzyme and SutA-σ^S-RANP complexes. a The structural comparison between SutA-RNAP-σ^S (closed lobe, green), SutA-RNAP-σ^S (open lobe, cyan), and RNAP-σ^S (gray) . **b** SutA-RNAP-σ^S (open lobe; colored as above) shows 15° opening of β gate loop and 5 Å increase in width of the RNAP main cleft compared with SutA-RNAP-σ^S (closed lobe; green). **c** The wedge loop of SutA invades into the gap of RNAP-β protrusion/lobe in SutA-RNAP-σ^S (open lobe). WL, wedge loop. **d** Structure modeling shows that the wedge loop of SutA encounters steric hindrance with the β lobe domain in closed conformation of SutA-RNAP-σ^S (closed lobe). **e** Superimposition of SutA-RANP-σ^S (open lobe), DksA/ppGpp-bound *E. coli* RNAP-rrnBP1 promoter complex (PDB: 7KHI, yellow), and TraR-bound *E. coli* RNAP closed complex (PDB: 6PSQ, blue).

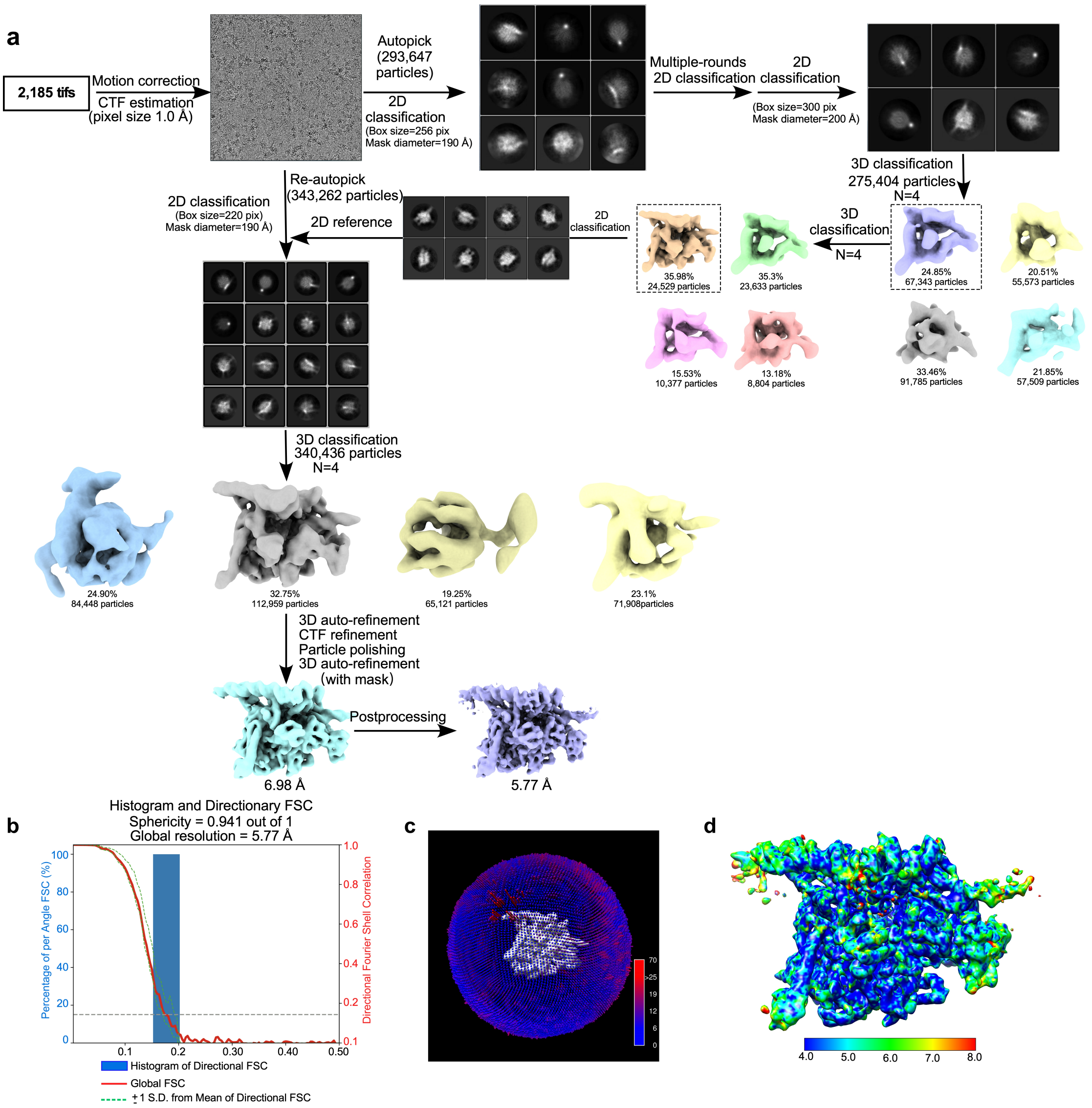

Supplementary Figure 6. The processing pipeline for cryo-EM map construction of *Pae* SutA-σ^S-RPo. a The flowchart of cryo-EM data process for SutA-σ^S-RPo. **b** The 3D FSC plot of the SutA-σ^S-RPo dataset. The dotted line represents the 0.143 FSC cutoff indicating a nominal resolution of 5.77 Å. **c** The angular distribution of SutA-σ^S-RPo single-particle projections colored by number of particles of each projection. **d** The local resolution of SutA-σ^S-RPo.

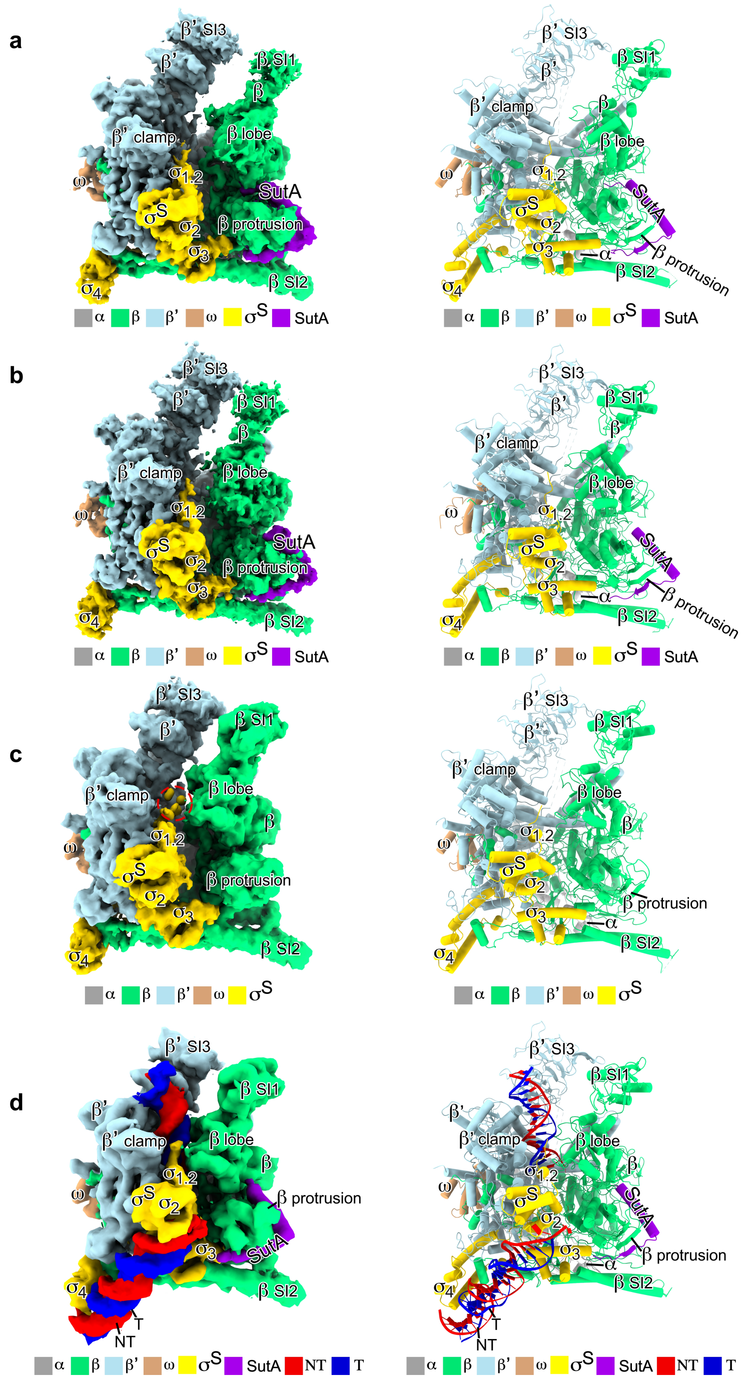

Supplementary Figure 7. The comparison of cryo-EM maps and structural models for (a) SutA-σ^S-RANP (open lobe), (b) SutA-σ^S-RANP (closed lobe), (c) *Pae* RANP-σ^S-holoenzyme, and (d) SutA-σ^S-RPo at the same view orientation.
